# Supplementary material for: Immunoinformatics-guided recombinant polypeptide-based enzyme-linked immunosorbent assay for seromonitoring of laboratory animals for minute virus of mice and Kilham rat virus
Source: PLoS One. 2024 Feb 27;19(2):e0298742. doi: 10.1371/journal.pone.0298742 (PMC10898725; doi:10.1371/journal.pone.0298742)
Supplement: S1 Fig — The panels contain the original gels, with the lanes used for the figure being labeled with the same numbers, whereas the rest of the lanes are designated as ‘x’. (PDF) [file pone.0298742.s001.pdf]

Original gel of figure 2a

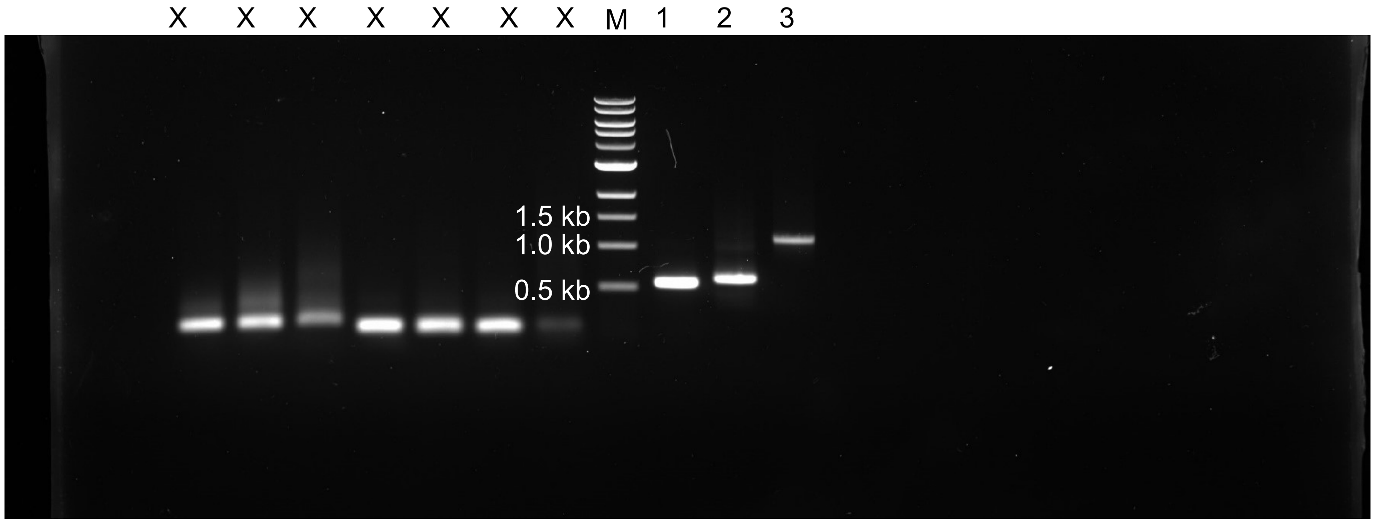

Shared Figure 2a

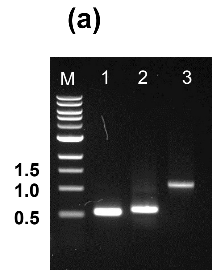

Cloning of CD. Fragments of C and D were amplified separately and then joined by SOE-PCR. M, DNA marker; 1, PCR amplified C gene fragment (528 bp); 2, PCR amplified D gene fragment (573 bp); 3, joined CD fragment (1.1 kb). The image was captured using Biorad Chemidoc imaging system.

Original gel of figure 2b

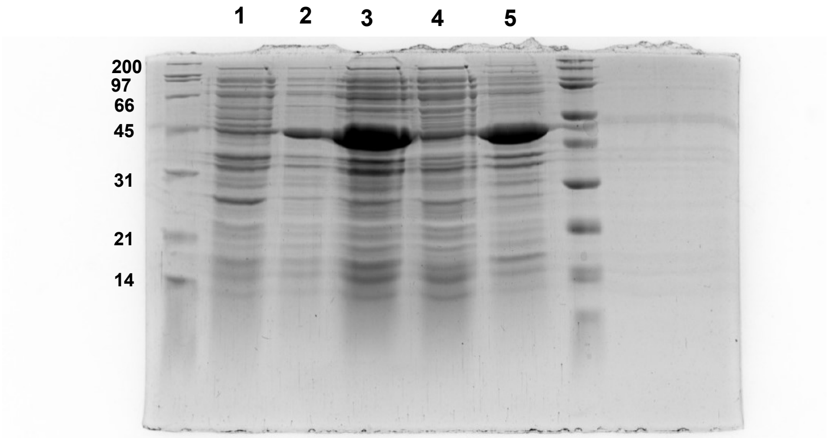

Shared Figure 2b

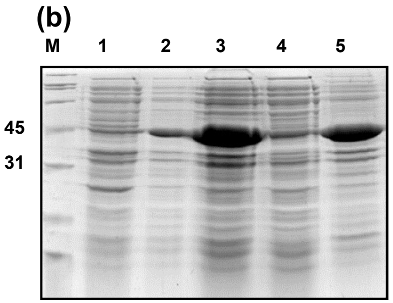

SDS-PAGE analysis of CD protein. Transformed BL21 cells induced with IPTG were separated into soluble and insoluble fractions, and subjected to SDS-PAGE. M, molecular weight marker (kDa); 1, total cell before induction; 2, total cell after induction; 3, total cell after sonication; 4, supernatant; 5, pellet solubilized in 8 M urea. The image was captured using Biorad Chemidoc imaging system.

Original chromatogram of Figure 2c

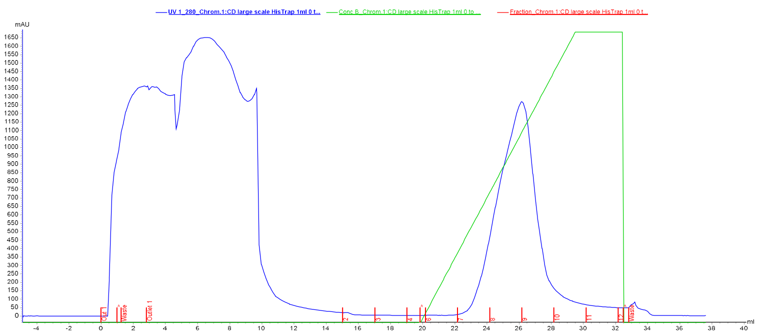

Original gel of figure 2d

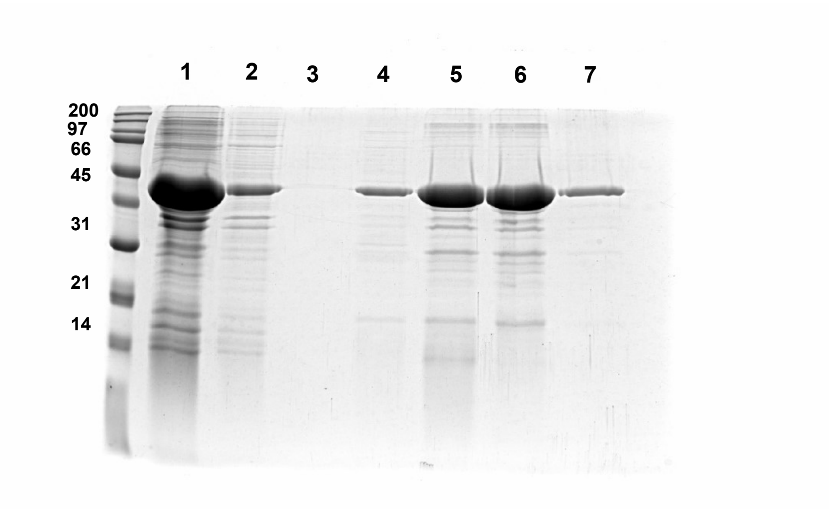

Shared Figure 2d

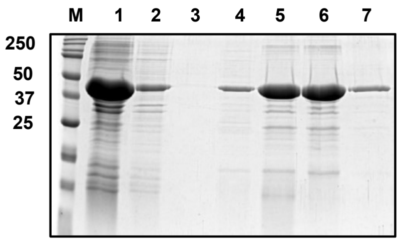

SDS-PAGE analysis of CD protein during purification. The proteins were visualized by Coomassie staining. M, molecular weight marker (kDa); 1, sample before loading on the column; 2, flow-through; 3-7, elution fractions after NiHP chromatography. The image was captured using Biorad Chemidoc imaging system.

Original gels of figure 2e

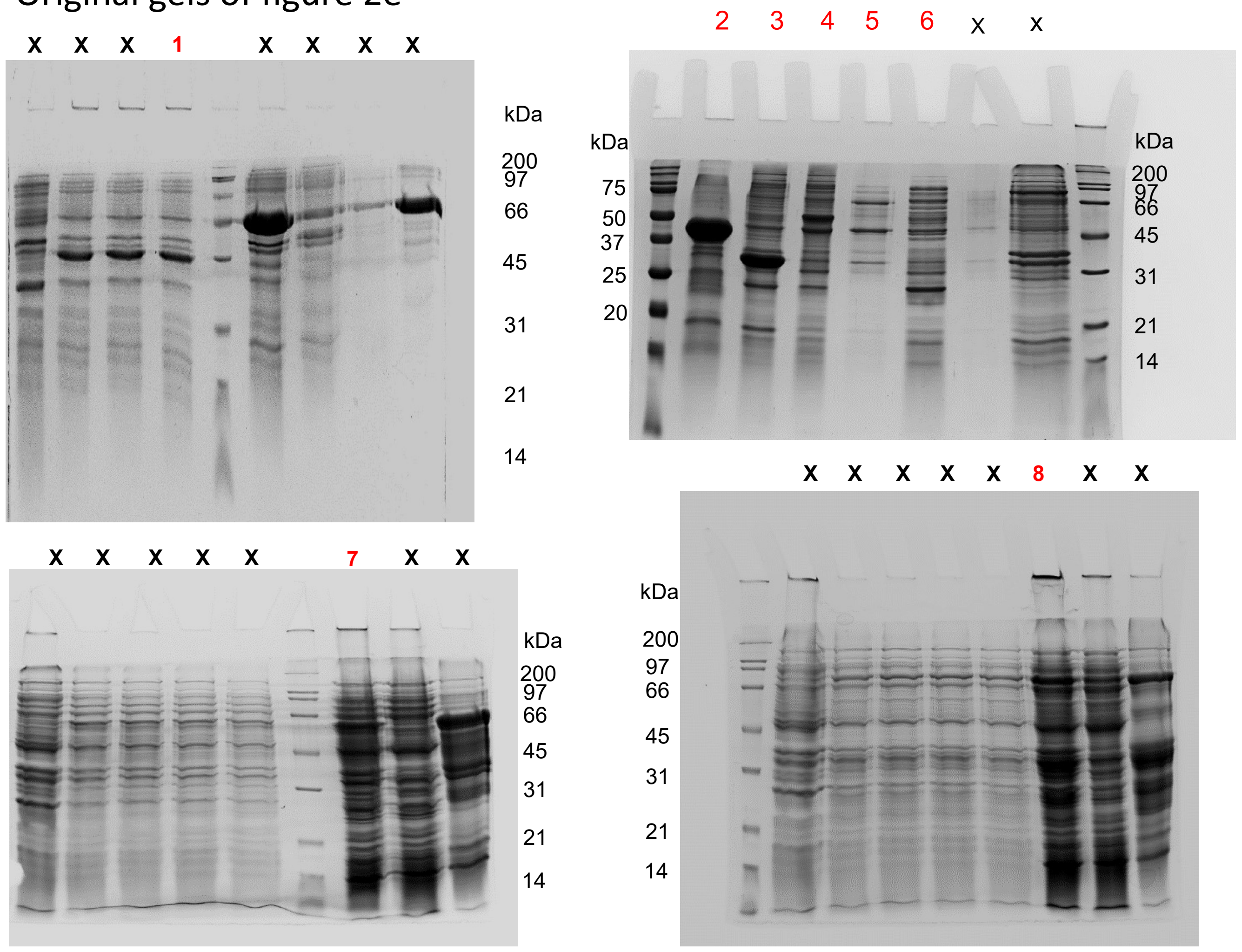

Shared Figure 2e

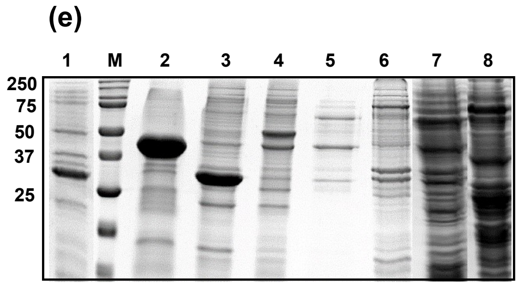

SDS-PAGE analysis of the expressed polypeptides. BL21 cells transformed with plasmids carrying the respective constructs were induced with IPTG, and the extracts were subjected to SDS-PAGE under reducing conditions, followed by Coomassie staining. 1, Polypeptide A; M, molecular weight marker (kDa); 2, Polypeptide CD; 3, Polypeptide E; 4, Polypeptide FG; 5, MVM VP2 protein; 6, MVM NS1 protein; 7, KRV VP2 protein, 8, KRV NS1 protein. The image was captured using Biorad Chemidoc imaging system.

Original blots for figure 2f

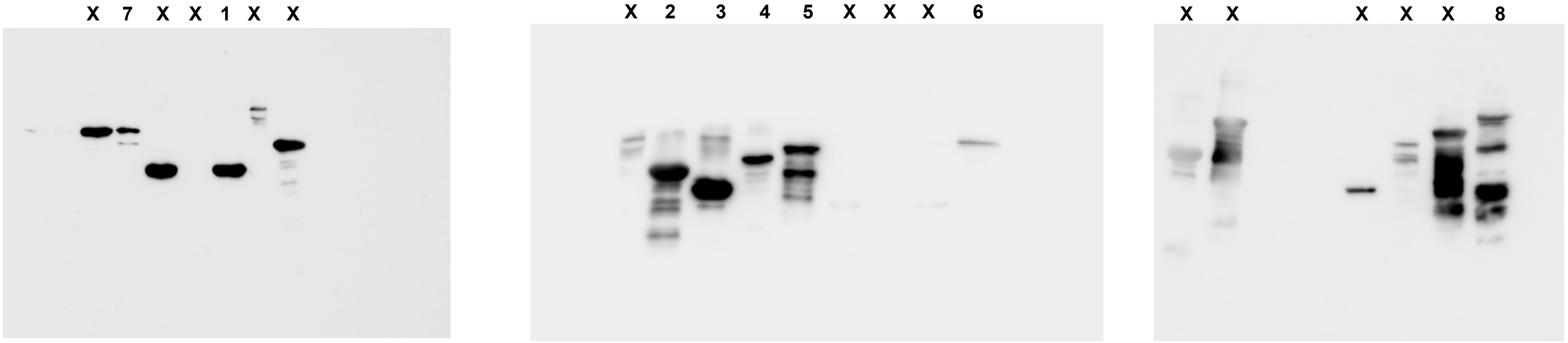

Shared blot for figure 2f

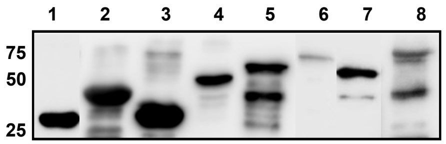

Western blotting. Proteins transferred onto PVDF membrane were probed using HRP-conjugated anti-His MAb. 1, Polypeptide A; M, molecular weight marker (kDa); 2, Polypeptide CD; 3, Polypeptide E; 4, Polypeptide FG; 5, MVM VP2 protein; 6, MVM NS1 protein; 7, KRV VP2 protein; 8, KRV NS1 protein. The position of three molecular weight markers (kDa) are shown on the left. The image was captured using Biorad Chemidoc imaging system.
