# Supplementary material for: Immunoinformatics-guided recombinant polypeptide-based enzyme-linked immunosorbent assay for seromonitoring of laboratory animals for minute virus of mice and Kilham rat virus
Source: PLoS One. 2024 Feb 27;19(2):e0298742. doi: 10.1371/journal.pone.0298742 (PMC10898725; doi:10.1371/journal.pone.0298742)
Supplement: S1 Table — (PDF) [file pone.0298742.s002.pdf]

Supplementary Table S1. Summary of the results of in-house ELISA against Xpress Bio ELISA kit for the detection of antibodies to MVM.

Serum No.	A	CD	A + CD	CD + VP2 FL	A + NS1 FL	VP2 FL	NS1 FL	NS1 + VP2	Kit	BSA
1	+	+	+	+	+	+	+	+	+	-
2	-	+	+	+	+	+	+	+	+	-
3	-	+	+	+	+	+	+	+	+	-
4	+	+	+	+	+	+	+	+	+	-
5	+	+	+	+	+	+	+	+	+	-
6	+	+	+	+	+	+	+	+	+	-
7	+	+	+	+	+	+	+	+	+	-
8	+	+	+	+	+	+	+	+	+	-
9	-	+	+	+	+	+	+	+	+	-
10	+	+	+	+	+	+	+	+	+	-
11	+	+	+	+	+	+	-	+	+	-
12	+	-	-	+	-	+	-	+	-	-
13	+	+	+	+	+	+	+	+	-	-
14	-	+	+	+	+	+	+	+	-	-
15	+	+	+	+	+	+	+	+	-	-
16	+	+	+	+	+	+	+	+	+	-
17	+	+	+	+	+	+	+	+	+	-
18	+	+	+	+	+	+	+	+	+	-
19	-	-	-	-	-	-	-	-	-	-
20	-	-	-	-	-	-	-	-	-	-
Pos.[a]	+	+	+	+	+	+	+	+	+	-
Neg.[b]	-	-	-	-	-	-	-	-	-	-
P/N[c]	14/6	17/3	17/3	18/2	17/3	18/2	16/4	18/2	14/6	0/20

[a] Positive control
[b] Negative control
[c] Total positive/total negative samples detected
