# Supplementary material for: Immunoinformatics-guided recombinant polypeptide-based enzyme-linked immunosorbent assay for seromonitoring of laboratory animals for minute virus of mice and Kilham rat virus
Source: PLoS One. 2024 Feb 27;19(2):e0298742. doi: 10.1371/journal.pone.0298742 (PMC10898725; doi:10.1371/journal.pone.0298742)
Supplement: S2 Table — (PDF) [file pone.0298742.s003.pdf]

Supplementary Table S2. Summary of the results of in-house ELISA against Xpress Bio ELISA kit the detection of antibodies to KRV.

Serum No.	E	FG	E + FG	FG + VP2 FL	E + NS1 FL	VP2 FL	NS1 FL	NS1 + VP2	Kit	BSA
1	+	+	+	+	+	+	+	+	-	-
2	-	-	-	-	-	-	-	-	-	-
3	+	+	+	-	+	-	-	-	-	-
4	-	+	+	+	+	-	-	-	-	-
5	-	-	-	-	-	-	-	-	-	-
6	-	-	-	-	-	-	-	-	-	-
7	-	-	-	-	-	-	-	-	-	-
8	-	-	-	-	-	-	-	-	-	-
9	-	-	-	-	-	-	-	-	-	-
10	+	+	+	+	+	+	+	+	+	-
11	+	+	+	+	+	+	+	+	+	-
12	-	-	-	-	-	-	-	-	-	-
13	-	-	-	-	-	-	-	-	-	-
14	+	-	+	+	+	+	-	+	-	-
15	+	+	+	+	+	+	+	+	+	-
16	+	+	+	+	+	+	+	+	+	-
17	+	+	+	+	+	+	+	+	-	-
18	-	-	-	-	-	-	-	-	-	-
Pos.[a]	+	+	+	+	+	+	+	+	+	-
Neg.[b]	-	-	-	-	-	-	-	-	-	-
P/N[c]	8/10	8/10	9/9	8/10	9/9	7/11	6/12	6/12	4/14	0/18

[a] Positive control
[b] Negative control
[c] Total positive/total negative samples detected
