# Supplementary material for: Immunoinformatics-guided recombinant polypeptide-based enzyme-linked immunosorbent assay for seromonitoring of laboratory animals for minute virus of mice and Kilham rat virus
Source: PLoS One. 2024 Feb 27;19(2):e0298742. doi: 10.1371/journal.pone.0298742 (PMC10898725; doi:10.1371/journal.pone.0298742)
Supplement: Attachment — Submitted filename: Response to Reviewers.docx [file pone.0298742.s004.docx]

**Response to Journal/Editor’s comments**

1. Please ensure that your manuscript meets PLOS ONE's style requirements, including those for file naming. The PLOS ONE style templates can be found at
   <https://journals.plos.org/plosone/s/file?id=wjVg/PLOSOne_formatting_sample_main_body.pdf> and 
   <https://journals.plos.org/plosone/s/file?id=ba62/PLOSOne_formatting_sample_title_authors_affiliations.pdf>

Response: The revised manuscript conforms to PLoS ONE’s style.

2. We suggest you thoroughly copyedit your manuscript for language usage, spelling, and grammar. If you do not know anyone who can help you do this, you may wish to consider employing a professional scientific editing service.

Response: The manuscript has now been copyedited through Grammarly software. 
Copies of the revised manuscript showing the changes as well as a clean copy are uploaded separately, as per the requirement of PLoS ONE.

3. Note from Emily Chenette, Editor in Chief of PLOS ONE, and Iain Hrynaszkiewicz, Director of Open Research Solutions at PLOS: Did you know that depositing data in a repository is associated with up to a 25% citation advantage (<https://doi.org/10.1371/journal.pone.0230416>)? If you’ve not already done so, consider depositing your raw data in a repository to ensure your work is read, appreciated and cited by the largest possible audience. You’ll also earn an Accessible Data icon on your published paper if you deposit your data in any participating repository (<https://plos.org/open-science/open-data/#accessible-data>).

Response: The original gels/blots are now uploaded as Supporting Information.

4. Thank you for stating the following financial disclosure:
   [This study was supported by a grant (BT/PR15810/ADV/90/198/2015) from the Department of Biotechnology, Ministry of Science and Technology, Government of India, to NRH and SGR.].  
   Please state what role the funders took in the study.  If the funders had no role, please state: ""The funders had no role in study design, data collection and analysis, decision to publish, or preparation of the manuscript."" 
   If this statement is not correct you must amend it as needed. 
   Please include this amended Role of Funder statement in your cover letter; we will change the online submission form on your behalf.

Response: The funders had no role and this is stated now in the cover letter.

5. Thank you for stating the following in the Acknowledgments Section of your manuscript:
   [This study was supported by a grant (BT/PR15810/ADV/90/198/2015) from the Department of Biotechnology, Ministry of Science and Technology, Government of India, to NRH and SGR.]
   We note that you have provided funding information that is not currently declared in your Funding Statement. However, funding information should not appear in the Acknowledgments section or other areas of your manuscript. We will only publish funding information present in the Funding Statement section of the online submission form. 
   Please remove any funding-related text from the manuscript and let us know how you would like to update your Funding Statement. Currently, your Funding Statement reads as follows: 
   [This study was supported by a grant (BT/PR15810/ADV/90/198/2015) from the Department of Biotechnology, Ministry of Science and Technology, Government of India, to NRH and SGR.]

Please include your amended statements within your cover letter; we will change the online submission form on your behalf.

Response: We have now included the amended funding statement in the cover letter. We thank you for taking up the responsibility of changing the online submission form.

6. PLOS ONE now requires that authors provide the original uncropped and unadjusted images underlying all blot or gel results reported in a submission’s figures or Supporting Information files. This policy and the journal’s other requirements for blot/gel reporting and figure preparation are described in detail at <https://journals.plos.org/plosone/s/figures#loc-blot-and-gel-reporting-requirements> and <https://journals.plos.org/plosone/s/figures#loc-preparing-figures-from-image-files>. When you submit your revised manuscript, please ensure that your figures adhere fully to these guidelines and provide the original underlying images for all blot or gel data reported in your submission. See the following link for instructions on providing the original image data: <https://journals.plos.org/plosone/s/figures#loc-original-images-for-blots-and-gels>.
     
   In your cover letter, please note whether your blot/gel image data are in Supporting Information or posted at a public data repository, provide the repository URL if relevant, and provide specific details as to which raw blot/gel images, if any, are not available. Email us at [plosone@plos.org](mailto:plosone@plos.org) if you have any questions.

Response: The original gels/blots are now uploaded as Supporting Information.

7. Please include captions for your Supporting Information files at the end of your manuscript, and update any in-text citations to match accordingly. Please see our Supporting Information guidelines for more information: <http://journals.plos.org/plosone/s/supporting-information>.

Response: Captions for Supporting Information files are now included at the end of the manuscript.

**Responses to Reviewers**

**Reviewer #1**

1. The study represents a significant technical innovation regarding the sensitivity of the ELISA method. However, as acknowledged by the authors themselves, additional experiments are necessary to assess both sensitivity and, importantly, specificity. The increasing diversity of rodent parvoviruses and their impact on research underscores the necessity for specificity tests.

   Another crucial consideration is the health status of the animals. Colonies maintained under specific-pathogen-free (SPF) conditions should be free of pathogens such as parvovirus. In this context, a more sensitive test may not significantly impact the outcome since detecting the virus in just a few sentinel animals (with no limitations on the quantity of samples) would suffice to trigger a report and initiate immediate colony cleaning procedures.

   On the other hand, ongoing experiments could benefit from a more specific and sensitive ELISA test to identify infected animals, particularly in subclinical conditions. Depending on the expected impact on the results, this could lead to the elimination or treatment of affected animals.

Response: We deeply appreciate these comments from the Reviewer. We have included some of this information in the penultimate paragraph of Discussion.

2. The methodology for in silico prediction, cloning, expression, purification, and evaluation is well-organized and thoroughly documented. However, the performance was compared to only one commercial kit for each virus. It would be valuable to compare it to other commercial kits as well.

Response: Comparison to only one commercial kit was due to the non-availability of other kits in India at the time the study was conducted. In addition, we had limitations on funds.

3. In line 278, FG + NS1 is not depicted in Fig. 3b and 4b, nor is it included in Table S1.

Response: We regret that there was an error in both the Figure and the Table. It should have been FG + VP2 FL and E + NS1 Fl. The fragment FG is part of NS1 itself, and hence tests have not been performed with FG + NS1 combination. These have now been corrected.

4. Basic statistical analyses are absent from Figures 3 and 4.

Response: We thank the reviewer for pointing this out. We are of the opinion that statistical analysis is inappropriate for individual samples as depicted in Fig. 3. However, analysis of values against that of the kit for the samples shown in Fig. 3a and 3b is now stated in the text.

On the other hand, significance analyses are now provided for various comparisons for Fig. 4a and 4b, where all samples are tested against the various antigens or combinations. Two tables showing the P values for the various comparisons are now included.

5. I recommend including specificity tests in the study.

Response: It is not clear to us whether the Reviewer means cross-reactivity or diagnostic specificity here. If it was the former, such tests are difficult to do without pathogen-specific antigens and antisera. While it may be possible to procure antisera, it is difficult to obtain pathogen-specific antigens, and in particular, recombinant antigens that are known to work in the format that we have applied. If the Reviewer was referring to diagnostic specificity, we would like to submit that the manuscript describes proof-of-concept and requires much more work to evaluate and validate diagnostic parameters (see response to Reviewer #2).

**Reviewer #2**

1. Although the study did not directly involve animal experimentation, the reporting should be generally based on the ARRIVE guidelines (Percie du Sert, N.; Hurst, V.; Ahluwalia, A.; Alam, S.; Avey, M.T.; Baker, M.; Browne, W.J.; Clark, A.; Cuthill, I.C.; Dirnagl, U.; et al.; The arrive guidelines 2.0: Updated guidelines for reporting animal research. PLoS Biol. 2020, 18, e3000410), since laboratory animal derived sample material was used.
   The authors should here especially comment on all measures regarding sample size calculation, randomization of samples and blinding.
   As this study aims to validate a diagnostic methods, inclusion and exclusion criteria of sample material should be discussed in detail and a statistical calculation of the results including estimation of the diagnostic sensitivity and specificity would be of value.

Response: We have considered reporting based on ARRIVE guidelines while formulating the manuscript. However, we would like to submit that this is a proof-of-concept study. In addition, ARRIVE guidelines are not mandatory to be followed in India, particularly for experimental research. Furthermore, the samples had been collected during an earlier study for the purpose of routine surveillance for pathogens at various laboratory animal colonies in India.

We would like to submit that our study is only a proof-of-concept study, and does not aim to validate an already developed method. Further development and validation would require power analysis for sample size calculation as well as randomization and blinding. Unfortunately, there is not enough data on the prevalence of laboratory animal pathogens in India to do a power analysis and calculate the sample size. Also, the study was conducted by using previously collected samples and hence it lacks randomization and blinding. This is noted in the last paragraph of Discussion as a limitation.

2. The authors used the novel ELISA on serum samples, which were re-used from a previous study. Can the authors comment on the storage conditions of the samples and potential influence on antibody stability?

Response: The samples had been stored at -80^o^C with very little freeze-thaw, and hence any instability of the antibodies can be ruled out.

3. The authors categorized samples in “positive” and “negative” based on results obtained by a commercial ELISA kit and re-tested them with the herein described ELISA. Since they tested four (mice) and 5 (rats) more samples positive compared to the commercial kit, they proposed that the novel ELISA would be “better” in terms of diagnostic sensitivity. How can the authors exclude, that the result from the novel method was false positive and lacks diagnostic specificity? Third-party testing (preferably with an alternative method) is strongly recommended to solve this discrepancy and draw proper conclusions. The number of analyzed negative samples should be generally increased to strengthen results to calculate the diagnostic test specificity (control for false positive test results).
   Furthermore, the results part should include a proper calculation of the diagnostic sensitivity and specificity of the assay.

Response: We completely agree with the Reviewer. We had been careful not to use the word ‘better’ in comparison with the commercial kit, and only stated that more samples were positive. We have still scrutinized the whole manuscript and avoided the use of the word ‘better.’ Also see response to #1 above.

As far as diagnostic sensitivity and specificity is concerned, and as stated above, these cannot be determined with this proof-of-concept study where sufficient number of samples were not used.

4. Line 342: reference 4 – Mähler, not Mahler

Response: We thank the reviewer for pointing this out. It has now been changed.
