# Supplementary material for: Immunoinformatics-guided recombinant polypeptide-based enzyme-linked immunosorbent assay for seromonitoring of laboratory animals for minute virus of mice and Kilham rat virus
Source: PLoS One. 2024 Feb 27;19(2):e0298742. doi: 10.1371/journal.pone.0298742 (PMC10898725; doi:10.1371/journal.pone.0298742)
Supplement: Attachment — Submitted filename: Response to Reviewers.docx [file pone.0298742.s005.docx]

We once again thank the reviewer and the editor for the comments. We have provided point-by-point responses below, and have made the necessary changes in the manuscript, including some other minor errors that we spotted during this revision.

**Response to Editor’s comments**

1. Authors should include in the manuscript that the present manuscript is a proof-of-concept study, and does not aim to validate an already developed method. Further development and validation would require power analysis for sample size calculation as well as randomization and blinding.

Response: This has been included in the last paragraph of the Discussion. This paragraph has also been revised for coherence and to avoid repetition of information.

**Response to Journal’s comments**

1. Please review your reference list to ensure that it is complete and correct. If you have cited papers that have been retracted, please include the rationale for doing so in the manuscript text, or remove these references and replace them with relevant current references. Any changes to the reference list should be mentioned in the rebuttal letter that accompanies your revised manuscript. If you need to cite a retracted article, indicate the article’s retracted status in the References list and also include a citation and full reference for the retraction notice.

Response: All the references are correct and none of them have been retracted.

**Responses to Reviewers**

**Reviewer #2**

1. Consider adding the statistical analysis also in the figure legend of Fig. 3.

Response: We thank the reviewer for this comment. Indeed, we considered adding statistical analysis for these samples while revising the manuscript which was submitted last. However, we feel that better inference can be drawn from statistical analysis on samples from a group of animals, particularly for diagnostic assays. It may be noted that analysis for individual samples may be more suited, for instance, for analysing immunogenicity of individual animals against an antigen. In order to keep the inference simple and meaningful in the context of a group of samples, we would not like to add statistical analysis for the data in Fig. 3.

2. "Table 5" is used for caption twice (one for p values of MVM and one for KRV).

Response: We apologize. It should have been Table 5 and Table 6. This has now been corrected both in the text and the table captions.

3. line 386 -392: Consider rephrasing

Response: We are unable to figure out what this refers to as there seems to be some discrepancy in the line numbers in document that the reviewer is seeing and the pdf document that was generated by the system. For example, line 401 as noted below is a blank line. However, we have rephrased what we think is a bit confusing and hope that it is clearer now. This is the second sentence in the 4^th^ paragraph in the Discussion (lines 374-376).

4. line 401: To to

Response: Thank you; this has been corrected. This in the 4^th^ line of the last paragraph of the Discussion (line 386).
